# Qingchang Suppository Ameliorates Colonic Vascular Permeability in Dextran-Sulfate-Sodium-Induced Colitis

**DOI:** 10.3389/fphar.2018.01235

**Published:** 2018-10-31

**Authors:** Boyun Sun, Jianye Yuan, Shiying Wang, Jiang Lin, Wanjun Zhang, Jiadong Shao, Ruiqing Wang, Bei Shi, Hongyi Hu

**Affiliations:** ^1^Department of Gastroenterology, China–Canada Center of Research for Digestive Diseases, Institute of Digestive Diseases, Longhua Hospital, Shanghai University of Traditional Chinese Medicine, Shanghai, China; ^2^Shanghai University of Traditional Chinese Medicine, Shanghai, China

**Keywords:** qingchang suppository, ulcerative colitis, epithelial permeability, vascular permeability, vascular endothelial barrier

## Abstract

Ulcerative colitis (UC), with a long course and repeated attack, severely affects patient's life quality and increases economic burden all over the world. However, the concrete causes and mechanisms of UC are still unclear, but it is generally considered that many factors participate in this process. Qingchang Suppository (QCS) has been used in treating rectitis and colitis for about 30 years in Shanghai, China. Its satisfactory clinical effects have been proved. The aim of this study is to investigate the effect and mechanisms of QCS on colonic vascular endothelial barrier in dextran sulfate sodium (DSS)-induced colitis. The results indicated that increased vascular permeability (VP) appeared earlier than increased intestinal epithelial permeability (EP) in the process of DSS-induced colitis. QCS attenuated colonic tissue edema, vascular congestion and inflammatory cell infiltration. QCS inhibited the elevation of MPO, TNF-α, and IL-6 levels in colon tissues and alleviated the microvascular damage induced by DSS. QCS also improved colonic hypoxia and decreased the expression of VEGF, HIF-1α, and iNOS. These results revealed that QCS can reduce colonic VP and can improve vascular endothelial barrier function maybe by regulating the VEGF/HIF-1α signaling pathway.

## Introduction

Ulcerative colitis (UC), a subtype of inflammatory bowel disease (IBD), is characterized by chronic inflammation of colonic mucosa and recurrent attack. Abdominal pain, diarrhea and mucopurulent bloody stools are the main clinical manifestations of UC (Floch, 2011). Its lesions mainly involve the mucous membrane and submucosa of the rectum, sigmoid, and descending colon, some parts of the transverse colon, or even the whole colon. Severe ulcers may invade the muscular layer and serosa, leading to perforation. UC with a wide range and >10 years is easy to develop into colorectal cancer (Masakazu, 2014). The World Health Organization has listed UC as one of the refractory diseases as it has a strong impact on quality of life due to its long course and repeated attacks. At present, the focus of UC treatment is to control mucosal inflammation and inhibit excessive immunoreactivity. Treatments with conventional drugs, including aminosalicylic acid, hormones, immunosuppressants, and biological agents, have been improved greatly in recent years.

The pathogenesis of UC is complicated, and so far, the exact etiology and pathogenesis remain unclear. UC is subject to immunological, mental, dietary, infectious, allergic, genetic and environmental factors. Thus, it is regarded as a multi-factorial disease resulting from interaction between host reactions, which are affected by immunity and heredity, and exogenous stimulation. Although many studies have reported the factors that may be involved in UC, no consensus has been reached on the primary cause of cell and tissue damage. It is disputed whether inflammation and the immune response are part of the initial damage or are secondary reactions. Intestinal epithelial barrier dysfunction is characterized by increased epithelial permeability (EP), which causes intraluminal bacteria and other antigens to traverse the epithelium into the mucosa, enhancing the immune response, triggering or aggravating UC (Mankertz and Schulzke, 2007; Alsadi et al., 2009). Most studies on the role of the epithelial barrier in the pathogenesis of UC have focused on increased EP (Alsadi et al., 2009; Jump and Levine, 2010). In fact, EP is not only determined by the epithelium, but also by the mucosal blood vessels. The vascular endothelial barrier plays an important role in keeping the integrity of the epithelium as it can maintain blood flow and deliver oxygen and nutrients to the epithelium, and prevent infiltration of inflammatory cells or proteins (Thornton and Solomon, 2002). Increased mucosal vascular permeability (VP) and reduced blood flow can cause intestinal tissue hypoxia (Taylor and Colgan, 2007), which induces increased expression of oxyradical or other inflammatory mediators, leading to intestinal epithelial cell damage and destruction of adjacent tight junctions (Rezaie et al., 2007). EP is increased when the integrity of the epithelial barrier is destroyed. Therefore, increased VP may be the initial factor in UC occurrence and recurrence, earlier than increased EP (Tolstanova et al., 2012). So, it is important to study the mechanisms involved in the increase of VP in the early phase of UC, which may be helpful for prevention and treatment.

The traditional Chinese medicine (TCM) Xilei San (Hao et al., 2014) is widely used to treat mucosal inflammation in China. Qingchang Suppository (QCS) (Dai et al., 2010) is a pure TCM preparation, composed of Indigo Naturalis, Herba Portulacae, Radix Notoginseng, Gallnut and borneol. It was first prescribed by Professor Ma Guitong, a renowned TCM doctor in China, based on Xilei San theory accumulated clinical practice. QCS has shown satisfactory clinical results in treating UC in recent years. It can clear away heat and toxic materials, promote blood circulation and remove blood stasis, and eliminate turbidity to promote tissue regeneration and ulcer recovery. As a suppository, it has the advantages of convenient use and easy absorption. This also confirms the fact that UC lesions are often located in the distal colon, sigmoid colon and rectum.

The aim of this study was to investigate the effect of QCS on colonic microvascular permeability in the onset and progression of DSS-induced colitis and the mechanisms involved.

## Materials and methods

### Herbs and reagents

QCS (Z05170722) was supplied by Longhua Hospital, Shanghai University of Traditional Chinese Medicine. It consisted of the following herbs: Radix Notoginseng, Indigo Naturalis, Gallnut, Herba Portulacae and borneol, in a ratio of 2:2:5:5:1. Sulfasalazine Suppository (SASP) is a yellow suppository which was made of 0.5 g sulfasalazine with fatty matrix. SASP was purchased from Shanxi Tongda Pharmaceutical Co. Ltd. (Shanxi, China). Dextran sulfate sodium salt (DSS) (MW 36,000–50,000) was purchased from MP Biologicals (Santa Ana, CA, USA). Indirubin, Notoginsenoside R1, Ginsenoside Rg1, Ginsenoside Rb1, Ginsenoside Re, and Luteolin were purchased from the National Institutes for Food and Drug Control (Shanghai, China). Kaempferol, Quercetin, Gallic acid, α-Linolenic acid, and Methyl gallate were purchased from Shanghai Macklin Biochemical Co. Ltd. HPLC grade acetonitrile was obtained from Fisher (Geel, Belgium) and HPLC grade formic acid from ANPEL Laboratory Technologies (Shanghai, China). Water was purified using a Milli-Q Academic System (Millipore, Billerica, MA, USA).

### Animals

Male Sprague-Dawley rats (170–200 g) were obtained from Shanghai Laboratory Animal Center (SLAC, Shanghai, China). All animals were housed in a specific pathogen-free laboratory in the Department of Laboratory Animal Science of Shanghai University of Traditional Chinese Medicine (Shanghai, China). Animals were maintained on a 12 h light/dark cycle under controlled temperature (22 ± 2°C) and humidity (50 ± 10%) with standard environmental conditions. All animals had unlimited access to standard diet and water. They were allowed to acclimatize for 7 d before use.

### Experimental design

This experiment includes two parts.

The first part was to confirm the sequence of VP and EP changes in experimental colitis by observing the dynamic changes of intestinal epithelial permeability and vascular permeability in consecutive 6 days (the day before oral administration of DSS, the first day, 2nd, 3th, 4th, 5th day with oral administration of DSS). In the first part, experimental rats were randomly divided into six groups: A: DSS treating 0 d; B: DSS treating 1d; C: DSS treating 2 d; D: DSS treating 3 d; E: DSS treating 4 d; F: DSS treating 5 d. Meanwhile, control rats were set up which only drink water instead of DSS, and rats were killed every day for DAI evaluation and for EP, VP observation. Group A had 10 rats and there were 8 rats in each other group. The rats were euthanized on day 1–5 after treatment with 5% DSS except that in the group A, which were given distilled water and were euthanized on the day before treatment with 5% DSS.

The second part was carried out to investigate the effect of Qingchang suppository (QCS) on the intestinal VP when it increased according to the results of the first part. In the second part, rats were randomly divided into six groups: control, model, QCS low dose (QCS-L), QCS medium dose (QCS-M), QCS high dose (QCS-H) and SASP. Each group had 8 rats. Rats were given 5% DSS water for 3 d except that in the control group, which were given distilled water. The rats in the QCS-L, QCS-M, QCS-H and SASP groups were treated with 0.36, 0.72 or 1.44 g/kg QCS or 0.135 g/kg SASP separately (The medium dose of QCS and the dose of SASP was defined according to human-rat equivalent dosage conversion. The low dose of QCS was defined as the half of the medium dose of QCS and the high dose of QCS was defined as the 2 times of the medium dose of QCS.) from day 1 to day 3 by rectal administration. In briefly, We used a flexible catheter to deliver the QCS and SASP 2 ml into rectum of rats when they were heated up to about 37°C and were turned into liquid. The rats in the control and model groups were treated with the same volume of distilled water by rectal administration. DSS solution was prepared freshly every day. The volume of DSS solution ingested by each rat was measured daily. Two centimeters of distal colon was removed for morphological examination, VP evaluation, tissue hypoxia detection, ultrastructural observation, and western blotting analysis. All the animal experiments were performed in accordance with the National Institutes of Health Guidelines for the Care and Use of Laboratory Animals and approved by the Animal Ethics Committee of Shanghai University of Traditional Chinese Medicine (No. SZY201608003).

### Identification of the chemical composition of QCS

#### Preparation of sample solutions

QCS (2.94 g) was mashed then extracted with 40 mL methanol by ultrasonication at room temperature for 2 h, and the supernatants were obtained by centrifugation at 5,000 rpm (Kubota 3740, Japan) for 20 min. Finally, the supernatants were filtered through a 0.22-μm membrane for LC–electrospray ionization (ESI)–tandem mass spectrometry (MS/MS) analysis.

#### HPLC

HPLC system (Shimadzu, Kyoto, Japan) consisted of an LC-30AD binary pump, DGU-14A degasser, SIL-30AC autosampler and CTO-30AC column oven. The mobile phase consisted of acetonitrile and water containing 0.1 (v/v) formic acid. The samples were analyzed on an Agilent ZORBAX SB-C18 column (1.8 m, 100 × 2.1 mm) with gradient elution system for 0–15 min, using 10–60% acetonitrile. The flow rate was 0.2 mL/min, and the column temperature was set at 45°C.

Each of the 11 reference compounds was accurately weighed and then dissolved in methanol to prepare the stock solutions of 1.0 mg/mL, and a diluted solution of each standard solution (100 ng/mL) was analyzed qualitatively and quantitatively. QCS (2.94 g) was mashed then extracted with 40 mL methanol by ultrasonication at room temperature for 2 h, and the supernatants were obtained by centrifugation at 5,000 rpm (Kubota 3740, Japan) for 20 min. Finally, the supernatants were filtered through a 0.22-μm filter membrane for LC-ESI-MS/MS.

#### LC-ESI-MS/MS

A triple quadrupole tandem mass spectrometer (Shimadzu) equipped with an ESI source interface was connected to the above Shimadzu HPLC system. Negative ion mode was preferred owing to its high sensitivity for analysis of most of the compounds. The optimized mass parameters were set as follows: collision energy, 35 V; nebulizing gas flow, 3.0 L/min; interface temperature, 300°C; drying gas flow, 10 L/min; DL temperature, 250°C; heat gas flow 10 L/min; heat block temperature, 400°C; Collision-Induced Dissociation gas, 230 kPa.

### Evaluation of disease activity index

Body weight, stool consistency, and occult blood or gross bleeding was scored each day during the experimental period. Disease activity index (DAI) was determined by calculating the mean of each score to evaluate the degree of colitis, as previously described (Murthy et al., 1993). DAI was calculated by grading on a scale of 0 to 4 using the following parameters: loss of body weight (0: normal; 1: 0–5%; 2: 5–10%; 3: 10–15%; 4: >15%); stool consistency (0: normal; 2: loose stools; 4: watery diarrhea); and occult blood (0: negative; 2: positive; 4: gross bleeding). The final result was expressed as the average of the three.

### Histopathology

Distal colon sections were fixed in 4% formaldehyde, dehydrated, and embedded in paraffin. The samples were sliced into 4 μm thick sections, then deparaffinized in xylene and were rehydrated in a decreasing concentration gradient of ethanol and were stained with hematoxylin and eosin (H&E). Colonic morphology was visualized using light microscopy with Olympus image analysis software (Olympus America, Melville, NY, USA). Colonic damage was assessed as described previously (Xiao et al., 2013). Briefly, each colon was scored considering (1) the severity of inflammation (0, none; 1, mild; 2, moderate; 3, severe); (2) the extent of inflammation (0, none; 1, mucosa; 2, mucosa and submucosa; 3, transmural); and (3) crypt damage (0, none; 1, 1/3 damaged; 2, 2/3 damaged; 3, crypt loss but surface epithelium present; 4, both crypt and surface epithelium lost). Scores were then added, resulting in a total histological score that ranged from 0 to 10.

### Quantitative evaluation of EP

We determined the plasma concentration of fluorescein isothiocyanate- conjugated (FITC)–dextran (MW 3.0–5.0 kDa; Sigma, St. Louis, MO, USA) after its intragastric administration to quantitatively evaluate the colonic EP. Before the experiments, rats were fasted for 18 h, but had unlimited access to water. Rats were given 20 mL/kg phosphate-buffered saline (PBS; pH 7.4) containing 22 mg/mL FITC–dextran by gavage 4 h before sacrifice. Blood samples were obtained by cardiac puncture and were centrifuged (3,000 rpm at 4°C for 20 min. Plasma (100 μL) concentration of fluorescein was measured using a Synergy H4 Hybrid Multi-Mode microplate reader (BioTeck, Winooski, VT, USA) with excitation wavelength of 485 nm and emission wavelength of 520 nm. Serially diluted samples of the marker were set as standards.

### Quantitative evaluation of VP

VP was determined as described previously (Tolstanova et al., 2009) with slight modification. Evans blue dye is a marker of albumin leakage since it binds tightly to albumin and it crosses the endothelial barrier as the complex Evans blue/albumin (MW 67 kDa). All rats were anesthetized with sodium pentobarbital and then received Evans blue (10 mg/kg) by tail vein injection 15 min before sacrifice. We measured EP and VP in the same animals. The distal colon was removed, cleaned, dried, and weighed. The colon samples were soaked in a centrifuge tube containing 2.0 mL formamide and the tube was placed in a thermostatic water bath. After incubation for 24 h at 60°C, the formamide extract was collected and the Evans blue concentration was measured by spectrophotometry at 610 nm. Results were expressed as mg dye/g dry weight of colon.

### Ultrastructural observation

Samples of colonic tissue were cut into three strips of 1 mm width, and were fixed in 2.5% glutaraldehyde immediately for 2 h at 4°C. After fully washing three times in 0.1 mol/L PBS, they were embedded in epoxy resin. The sliced sections (0.5–1 mm) were stained with toluidine blue for 30 s and washed in 0.1 M PBS. Thin sections (75 nm) from the selected area were stained with uranyl acetate and lead citrate and viewed under a H-600 transmission electron microscope at 80 kV (Hitachi, Tokyo, Japan).

### Detection of tissue hypoxia

Colonic tissue hypoxia was detected using the Hypoxyprobe-1 Omni Kit (Natural Pharmacia International, Burlington, MA, USA) as previously described (Tolstanova et al., 2012). Rats were anesthetized with sodium pentobarbital. Pimonidazole-HCl (60 mg/kg) was injected intravenously 90 min before autopsy after 5% DSS treatment, vehicle (water) ingestion or drug treatment. The removed distal colon (2 cm) was fixed in 4% formaldehyde for 24 h, dehydrated, and embedded in paraffin. The paraffin-embedded colon sections (5 mm thick) were deparaffinized, hydrated, blocked with 3% H_2_O_2_/water, and subjected to microwave antigen retrieval using a Dako target retrieval solution (Dako, Carpinteria, CA, USA). After overnight incubation with affinity-purified rabbit anti-pimonidazole antibody (PAb2627AP) at 4°C, they were incubated with anti-rabbit polymer horseradish peroxidase (Dako), with diaminobenzidine used as a peroxidase chromogen. We used hematoxylin for counterstaining and five fields from each slide were randomly selected, viewed and imaged under a fluorescence microscope (Nikon TE2000-U, Nikon, Japan), and analyzed using the Image Pro-Plus 6.0 software (Media Cybernetics, Silver Spring, MD). The minimal pixel was set at 50 pixels, and the final result was expressed as integral optical density (IOD)/ area of effective statistical.

### Western blotting

Distal colon tissue was homogenized in RIPA buffer (P0013B; Beyotime, Hangzhou, China) with phosphatase inhibitors (S1873; Beyotime), and protein concentration was determined using the bicinchoninic acid assay method. 20 micrograms of total protein that was extracted from 100 mg colonic mucosa was separated on 10% SDS-PAGE and transferred to polyvinylidene difluoride membranes (Millipore). After the membranes were blocked with 5% skimmed milk in Tris buffer saline–Tween 20 (TBST), membranes were immunoblotted with the primary antibody against vascular endothelial growth factor (VEGF) (ab46154; Abcam, Shanghai, China), hypoxia-inducible factor (HIF)-1α (ab2185; Abcam), inducible NO synthase (iNOS) (ab49999; Abcam) or β-actin (Huaan Biological Technology, Hangzhou, China). The primary antibodies were visualized with goat anti-rabbit peroxidase-conjugated antibody (Cell Signaling Technology, Danvers, MA, USA) using an enhanced chemiluminescence detection system (Millipore). The images of blots were acquired by the GBOX Chemi XT4 System (Syngene, Cambridge, UK) and GeneTools software (Syngene) was used for semi-quantitative analysis.

### Statistical analysis

SPSS version 16.0 (SPSS, Chicago, IL, USA) and GraphPad Prism 5 (La Jolla, CA, USA) were used for data analysis. Each value was expressed as mean ± *SD*. Differences between two groups were analyzed using the Student's *t*-test, and statistical comparisons among more than two groups were carried out by One-way analysis of variance (ANOVA) followed by Dunnett's test. *P* < 0.05 was considered statistically significant.

## Results

### Characterization and quantification of main biochemical components in QCS

**Figure 1:**
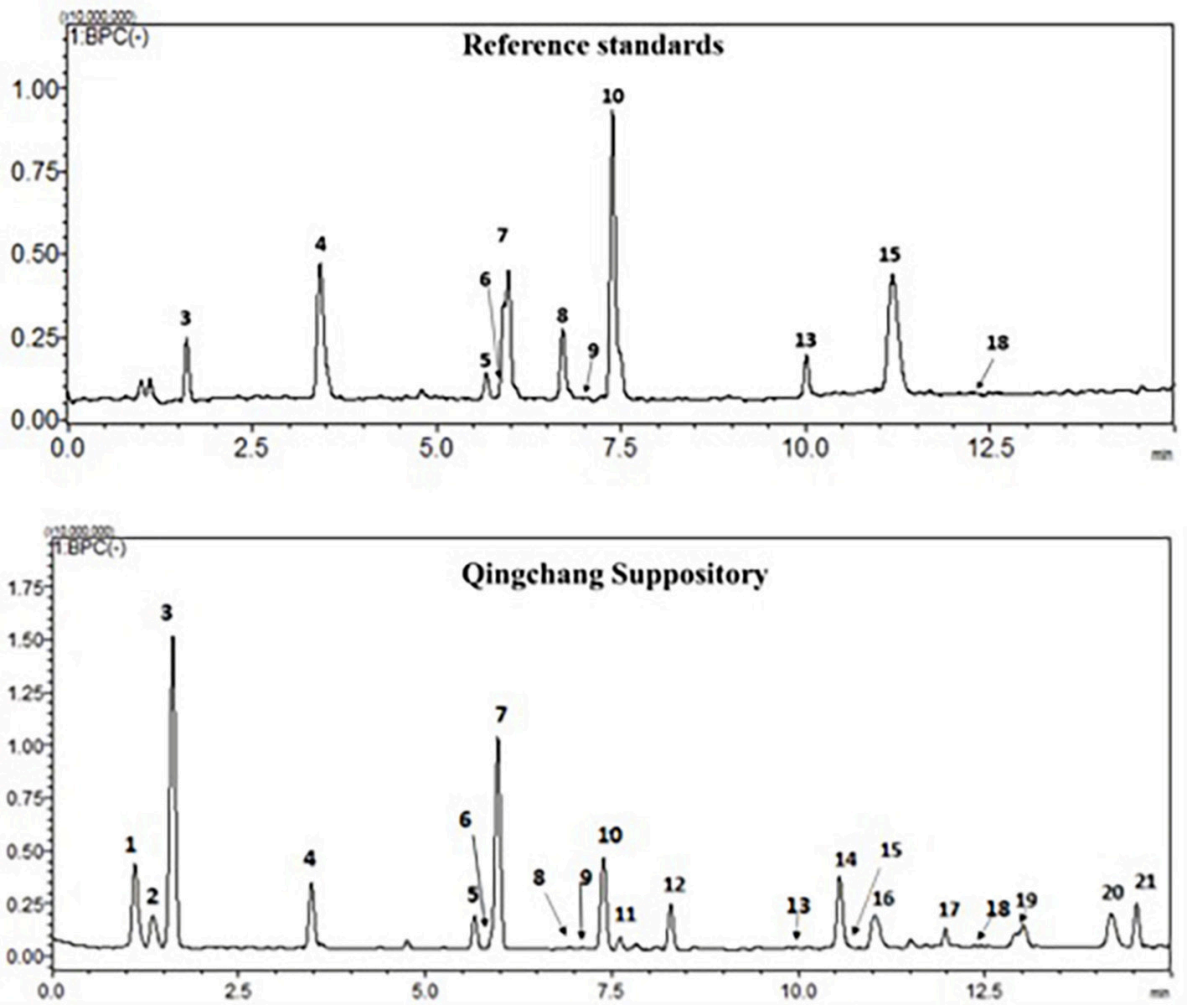
Typical chromatogram of QCS in negative ion mode by LC-ESI-MS.

The chemical profiles of QCS were analyzed using LC-ESI-MS/MS. In the typical base peak chromatogram (BPC) of QCS samples (Figure 1), 21 major peaks were detected and quantified (Supplementary Table 1). Eleven compounds (3–10, 13, 15, and 18) were unambiguously assigned by comparing with the respective reference standards, and five compounds (1, 2, 11, 12, and 21) were tentatively identified according to the MS/MS fragmentation patterns as well as information from the literature (Xin et al., 2008; Xu et al., 2012).

### Dynamic pathological changes of distal colon in DSS models of UC

**Figure 2:**
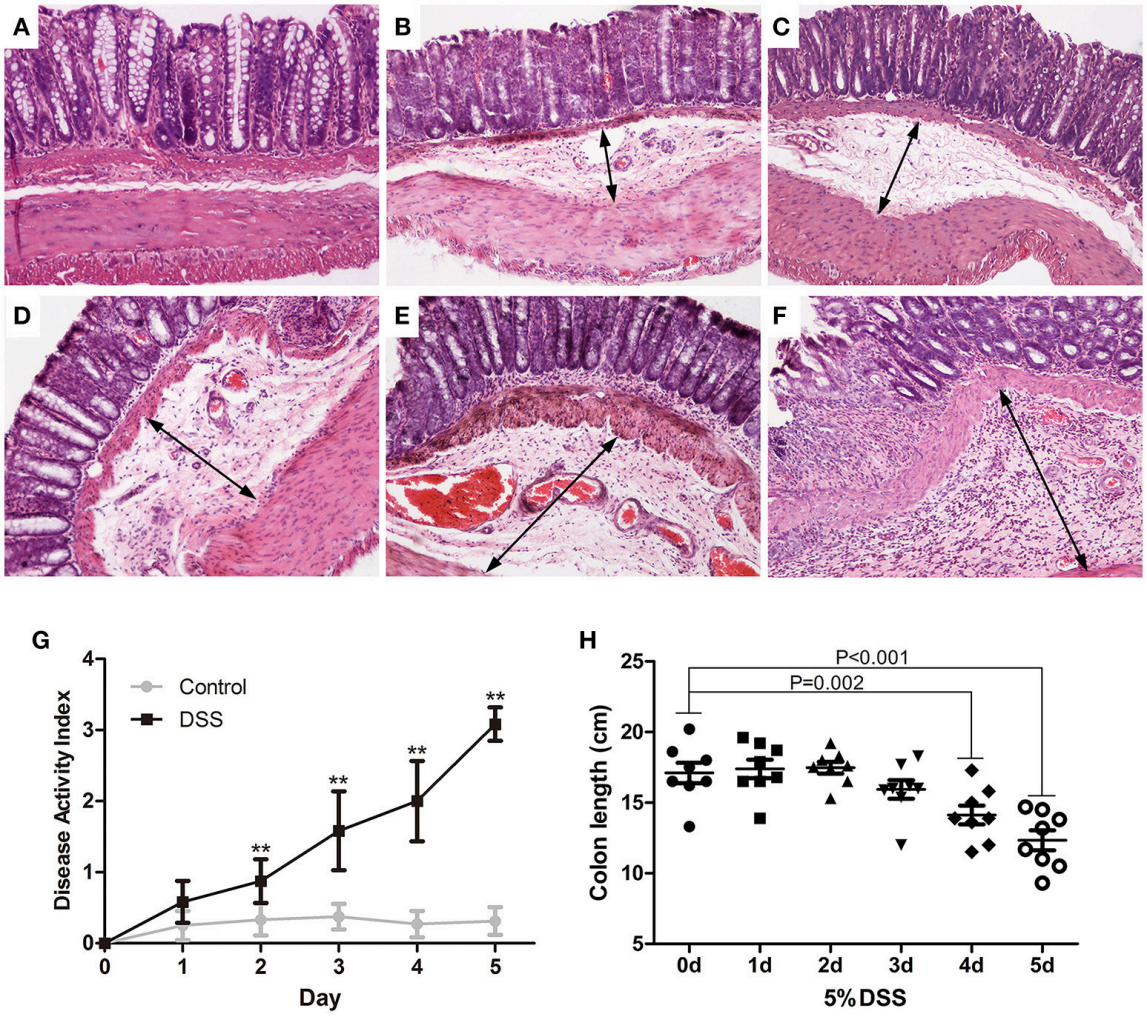
Dynamic pathological changes of the distal colon in DSS models of UC. Paraffin sections of the distal colon were stained with HandE (**A**: DSS 0 d; **B**: DSS 1d; **C**: DSS 2 d; **D**: DSS 3 d; **E**: DSS 4 d; **F**: DSS 5 d) (HandE staining, × 100). DAI **(G)** was assessed daily and colon length was measured on day 5 **(H)**. Data were expressed as mean ± *SD* (*n* = 8–10), ^*^P < 0.05, ^**^P < 0.01 vs. control group or vs. 0 d group.

The paraffin sections of colon were stained with H&E. Rare obvious histological changes were observed in the distal colon on day 0 (Figure 2A), followed by extensive submucosal edema at 24 h (Figure 2B) after induction of DSS colitis, and focal lamina propria inflammation on day 4 (Figure 2E) when the surface epithelial cell layer was still intact (Figures 2B–E). The blood vessels in the lamina propria and submucosa were dilated on day 4 (Figure 2E) after DSS ingestion. On day 5 after initiation of DSS treatment, the partial mucosal epithelium disappeared, and inflammatory cells increased and accumulated in the submucosa as well as the lamina propria (Figure 2F). DSS-induced colitis is characterized as a marked decrease in colon length (Xiao et al., 2013). After DSS administration, the DAI increased gradually (Figure 2G). Starting from day 4 (14.13 ± 1.78), DSS induced a rapid decrease in colon length (on day 5, 12.46 ± 1.77) (Figure 2H) and serious clinical symptoms (diarrhea and fecal blood).

### Increased colonic VP and EP in DSS-induced colitis

**Figure 3:**
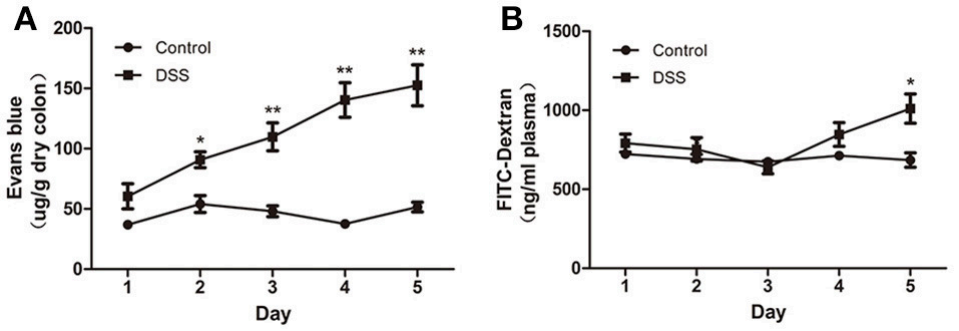
DSS administration increased colonic VP that preceded increased colonic EP in rats. **(A)** Quantitative measurement of VP by extracting extravasated Evans blue in the colonic mucosa. **(B)** Quantitative measurement of colonic epithelial permeability by determining serum concentration of FITC–dextran. Both VP and EP in the same animal were measured. *n* = 8. ^*^P < 0.05; ^**^P < 0.01 vs. control group.

The blue color was restricted to the distal colon. No apparent morphological changes were present (data not shown) on day 1 and there was no significant difference between the water and DSS-treated rats. On day 2, the concentration of Evans blue dye in tissue extracts of DSS-treated rats was significantly higher than that in the control rats, and it gradually increased from day 2 to day 5 (Figure 3A). In contrast to the increased content of Evans blue in colonic tissue, VP and plasma concentration of FITC–dextran were not obviously changed during the first 4 d but were significantly increased on day 5 after DSS treatment (Figure 3B). These results confirmed that increased colonic VP preceded increased EP after DSS treatment. In other words, the colonic vascular endothelium injury occurred earlier than colonic epithelial barrier disruption in DSS-induced colitis.

### QCS attenuated DSS induced colitis

**Figure 4:**
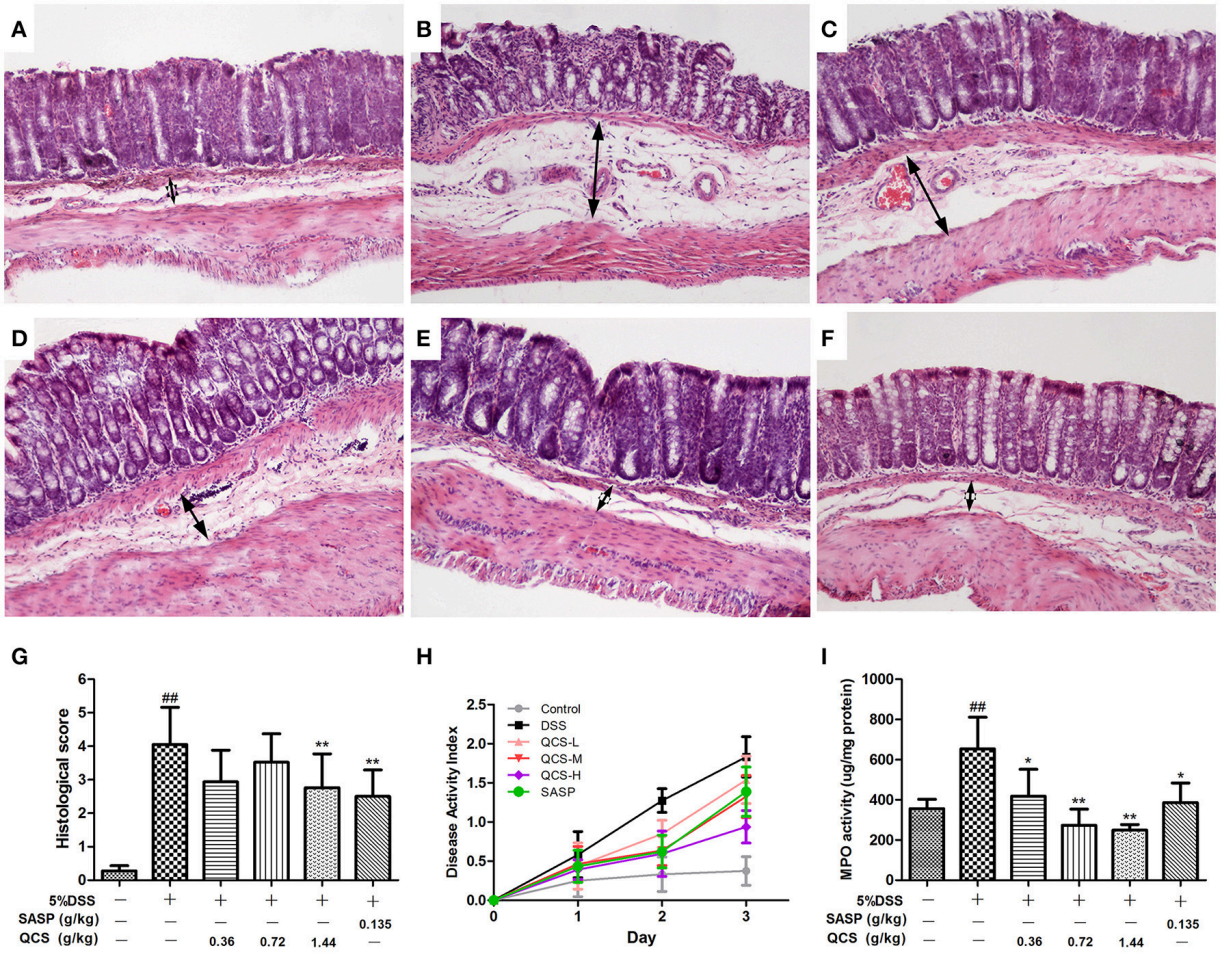
Effects of QCS on histopathological changes, DAI and MPO activity in colon of rats with DSS-induced colitis. **(A)** Control; **(B)** DSS model; **(C)** QCS 0.36 g/kg; **(D)** QCS 0.72 g/kg; **(E)** QCS 1.44 g/kg; **(F)** SASP 0.135 g/kg; (HandE staining, × 100); **(G)** histological score; and **(H)** The disease activity index (DAI) was determined by combining scores of body weight loss, stool consistency, and occult blood, The final result was expressed as the average of the three. **(I)** MPO activity. DSS administration was performed in all groups except the control group. QCS and SASP were administered to rats each day after DSS treatment. All rats were killed on day 3 after DSS administration, three sections from each animal tissue were scored, colonic tissue damage was evaluated by histopathological analysis (HandE staining). MPO activity in colonic tissue was determined. Data were expressed as mean ±*SD* (*n* = 8), ^*^P < 0.05, ^**^P < 0.01 vs. model group; ^##^*P* < 0.01, vs. control group.

To confirm the effect of QCS on colonic VP in rats, early colitis was induced by continuous oral administration of 5% DSS in drinking water for 3 d. At the same time, QCS and SASP were administered to rats each day. As expected, DSS induced colonic tissue damage, including the distorted arrangement of cells in crypts, extensive submucosal edema, dilated blood vessels in the submucosa, and little inflammatory cell infiltration (Figures 4A,B). On the contrary, the colonic damage in rats treated with QCS-H and SASP, but not QCS-L or QCS-M, were lessened (Figures 4C–F). The histological scores of the QCS-H group (2.76 ± 0.94) and the SASP group (2.50 ± 0.74) were significantly reduced, compared to the DSS model group (4.05 ± 1.04) (Figure 4G). But there was no significant difference in DAI scores among groups (Figure 4H). In addition, as another important symptomatic parameter in DSS induced colitis, colonic MPO activity, was increased in the DSS model group (654.07 ± 88.36) and was significantly suppressed by QCS (QCS-L group: 417.97 ± 62.27; QCS-M group: 273.03 ± 53.61; QCS-H group: 249.30 ± 24.69;) in a dose-dependent manner (Figure 4I).

### QCS attenuated DSS induced VP increase in colon

**Figure 5:**
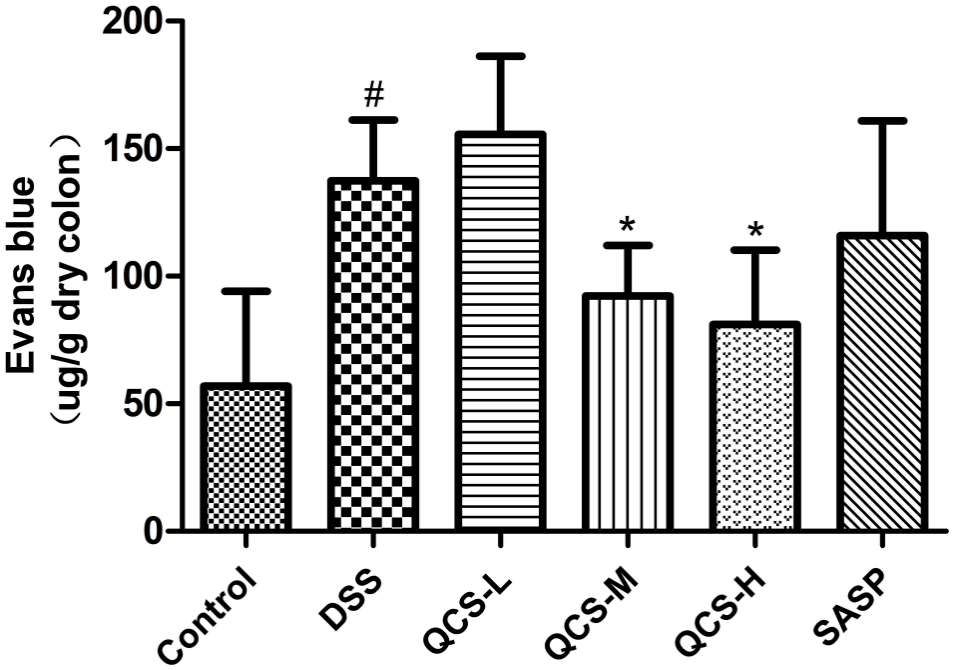
Effects of QCS on VP in colon of rats with DSS-induced colitis. Quantitative measurement of VP. Data were expressed as mean ±*SD* (*n* = 8), ^*^P < 0.05, ^**^P < 0.01 vs. model group; ^##^*P* < 0.01, vs. control group.

To evaluate the effect of QCS on VP, Evans blue concentration in colon tissue in each group was determined. Evans blue concentration in the model group (137.49 ± 21.26) was significantly higher than that in the control group (56.94 ± 5.51) (Figure 5). Compared with the model group, the colonic concentration of Evans blue was decreased in the rats treated with QCS at different doses. Moreover, concentration of Evans blue in the colonic tissues of all the treatment groups (QCS-M group: 92.20 ± 13.74; QCS-H: 81.9 ± 23.31) was significantly lower than that in the model group, except for the QCS-L group (155.63 ± 27.35) and SASP group (115.91 ± 19.26). These results suggest that QCS restore VP to improve tissue hypoxia.

### Effect of QCS on ultrastructural pathological changes in colonic tissue

**Figure 6:**
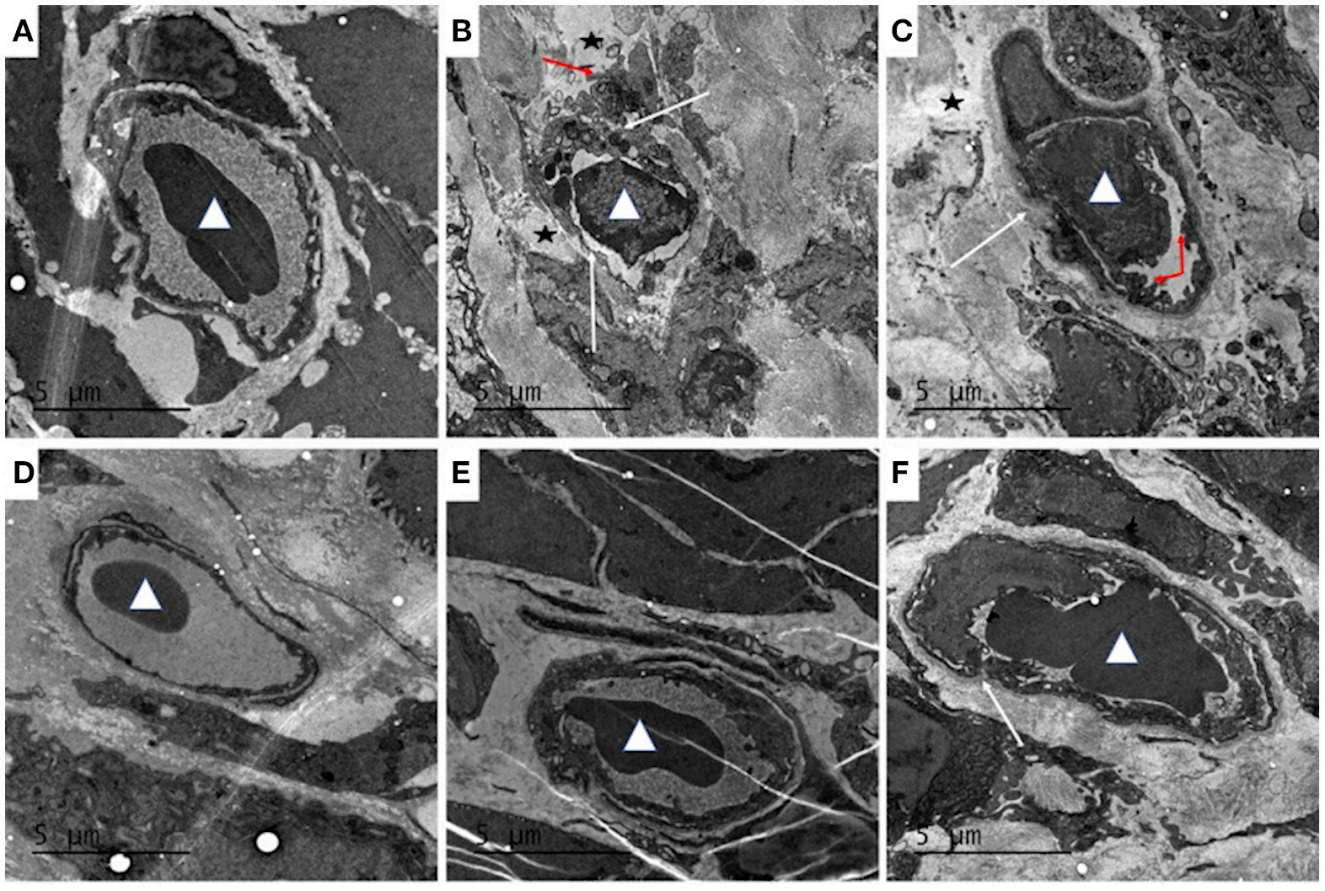
Effects of QCS on DSS-induced ultrastructural pathology changes in colon tissue. Histopathological examination of colon tissues by TEM (4,200 ×), *n* = 5. **(A)** Control; **(B)** DSS model; **(C)** QCS 0.36 g/kg; **(D)** QCS 0.72 g/kg; **(E)** QCS 1.44 g/kg; **(F)** SASP 0.135 g/kg. Red blood cells (white triangles). Platelet aggregation (red arrow). The microvascular basement membrane and endothelium (white arrow).

Transmission electron microscopy showed that the structure of the colonic vascular endothelium in the control group was intact, and the cells had normal morphology (Figure 6A). In the model group, the colonic vascular endothelium was swollen; the intercellular spaces were widened and the cells were ruptured; the basement membrane was not intact; and a large number of platelets aggregated in the vascular endothelium, with signs of some leakage. There was also perivascular edema and a large amount of fibrin deposition that likely resulted from vascular rupture (Figure 6B). The QCS-L group showed small breaks in the endothelial lining and platelets adhering to endothelial cells, with some extravascular platelets, but a decrease in fibrin deposition (Figure 6C). QCS-M and QCS-H groups showed reduced edema around the blood vessels and intact vascular basement membrane with no obvious platelet aggregation (Figures 6D,E). However, in the SASP group, a large number of red blood cells were clustered in the colonic vasculature, and increased micropinocytotic vesicles, swelling of endothelial cells and apoptosis, and perivascular edema were found (Figure 6F).

### Effect of QCS on colonic tissue hypoxia

**Figure 7:**
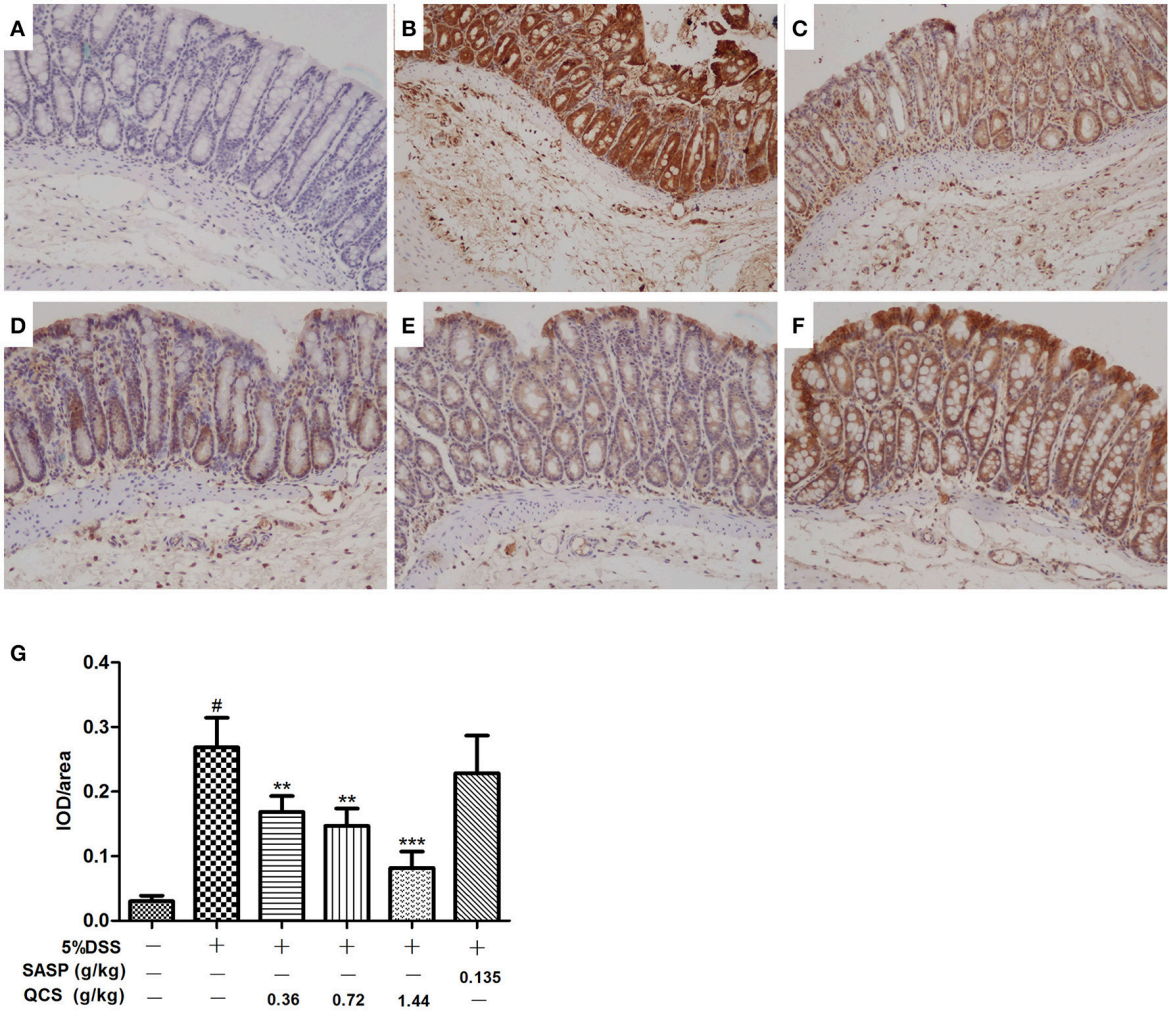
Effects of QCS on DSS-induced colon tissues hypoxia. Visualization of hypoxia in colonic mucosa by Hypoxyprobe-1 staining. Images are representative of three tissue slices (brown staining, × 100). **(A)** Control; **(B)** DSS model; **(C)** QCS 0.36 g/kg; **(D)** QCS 0.72 g/kg; **(E)** QCS 1.44 g/kg; **(F)** SASP 0.135 g/kg. **(G)** Quantified data of hypoxia in colonic mucosa. The final result was expressed as integral optical density (IOD)/ area of effective statistical. (*n* = 6/group). ^**^P < 0.01; ^***^P < 0.001, vs. model group; ^#^*P* < 0.05, vs. control group.

No significant hypoxia was observed in colonic tissue of the control group (Figure 7A). Conversely, colonic epithelium showed severe hypoxia, which extended to the lamina propria and submucosa in the model group (Figure 7B). Compared with the model group, the hypoxic state of the colonic tissue was significantly reduced in the QCS groups (Figures 7C–E), and was especially in the QCS-H group (Figure 7E). However, there was still severe hypoxia in the colonic mucosa of the SASP group (Figure 7F). Quantified data of hypoxia in colonic mucosa were measured (Figure 7G). These results suggest that QCS can improve hypoxia in colonic tissue induced by DSS.

### QCS inhibited DSS induced TNF-α and IL-6 production and expression of VEGF, HIF-1α and iNOS in colonic tissue

**Figure 8:**
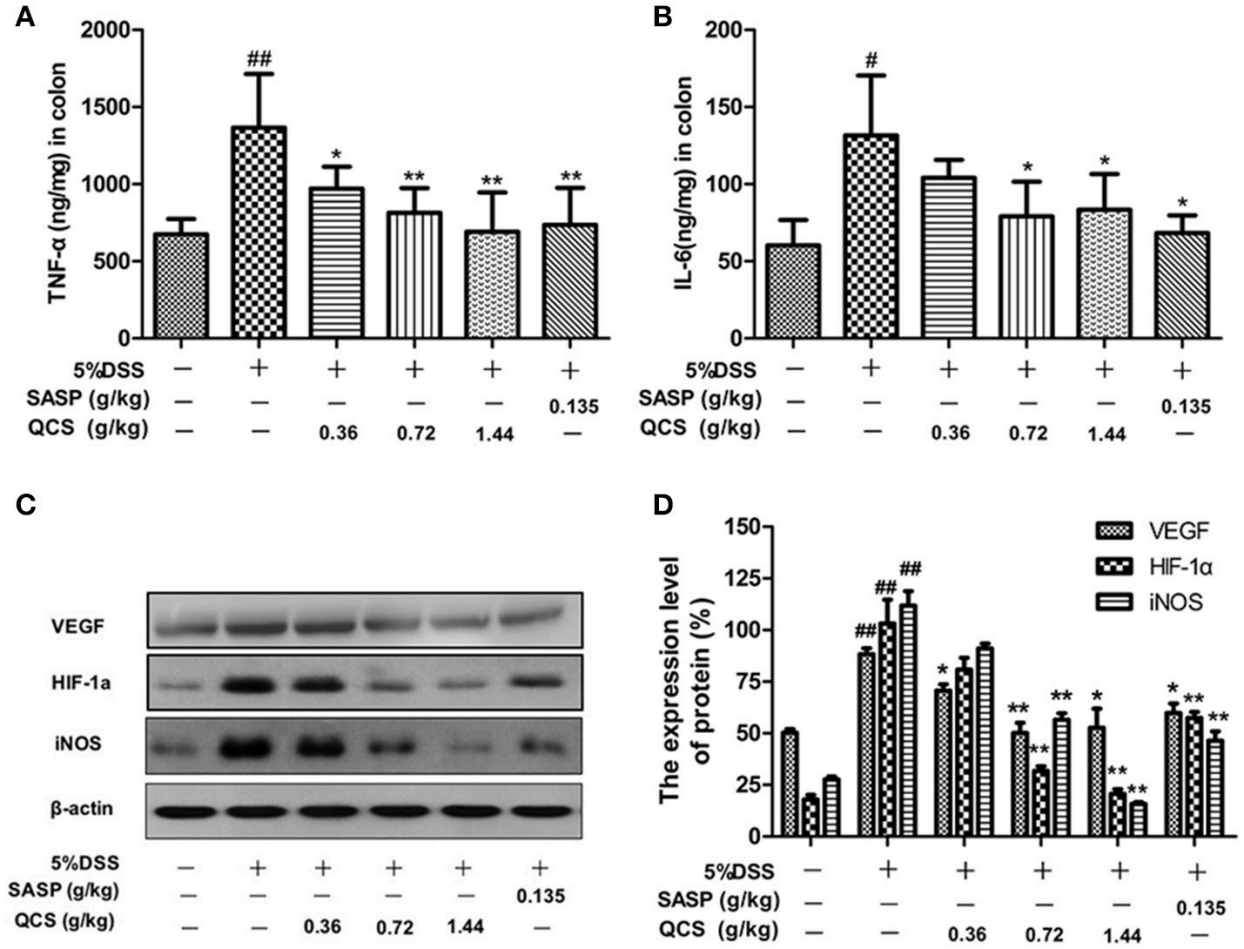
Effects of QCS on DSS-induced production of TNF-α and IL-6 and expression of VEGF, HIF-1α and iNOS in colonic tissue. **(A)** Concentration of TNF-α in colonic tissue; **(B)** concentration of IL-6 in colonic tissue. **(C,D)** Protein expression of VEGF, HIF-1α, and iNOS. β-Actin levels were used as loading controls. Results are expressed as mean ±*SD*; *n* = 5, ^*^P < 0.05; ^**^P < 0.01, vs. model group; ^#^*P* < 0.05; ^##^*P* < 0.01, vs. control group.

The levels of TNF-α and IL-6 in colonic tissue of the model rats (1182.6 ± 148.56, 131.63 ± 25.73) were significantly higher than those in the control rats (790.27 ± 30.06, 60.30 ± 10.18) (Figures 8A,B). However, treatment with different doses of QCS and SASP suppressed the increased levels of TNF-α (QCS-L group: 947.54 ± 23.63; QCS-M group: 874.51 ± 21.38; QCS-H group: 852.87 ± 22.44; SASP group: 854.51± 25.66) and IL-6 (QCS-M group: 79.01 ± 12.56; QCS-H group: 83.37 ± 10.17; SASP group: 68.29 ± 8.49) in DSS-induced colitis. These results suggest that QCS has anti-inflammatory effects. Expression of VEGF, HIF-1α, and iNOS in model group (88.30 ± 4.08; 103.16 ± 23.14; 111.78 ± 9.96) rats was substantially higher than that in the control group (50.31 ± 2.42; 17.96 ± 4.41; 27.69 ± 2.73) (Figures 8C,D). In contrast, expression of VEGF, HIF-1α and iNOS was inhibited in the QCS- and SASP-treated groups, especially in the QCS-H group (52.75 ± 13.06, 20.72 ± 4.46, 15.79 ±1.61). These results indicate that QCS maybe restores VP by suppressing the activation of the VEGF/HIF-1α signaling pathway.

## Discussion

Three major findings were extracted from this study: (1) vascular endothelial injury of UC occurs earlier than colonic mucosal dysfunction; (2) local tissue hypoxia may induce or aggravate intestinal epithelial barrier dysfunction; and (3) QCS may prevent the development of UC by improving damage to the colonic vascular endothelium to ameliorate tissue hypoxia, and by anti-inflammatory activity.

The chronological changes of DSS-induced colitis were categorized into two phases (Saijo et al., 2015); namely the early phase (days 2–3) and late phase (days 4–5). Hematochezia, as a primary clinical symptom, presents throughout the development of UC. Studies on rats have shown that hematochezia and weight loss occur on days 3–4 after DSS administration, but DSS directly penetrates the lamina propria at 24 h after DSS administration. Vascular smooth muscle and endothelial cells are attacked by DSS directly or indirectly through histamine regulation (Johansson et al., 2010). The results of histomorphological observation and EP determination confirmed that no significant change in intestinal epithelial structure was found in the early stage of DSS-induced colitis. It is further suggested that vascular injury occurs earlier than destruction of intestinal epithelial structure in the early stage of UC. Moreover, the indirect effect of histamine may be attributed to the increased expression of mast cells in the lamina propria (Kurashima et al., 2012), which also provides evidence for the early colon tissue edema of DSS-induced colitis.

An ischemic colitis model in mice showed massive bleeding and intestinal epithelial cell exfoliation after ligation of the distal colon artery for 3 d (Irkorucu et al., 2008). Experimental vascular congestion induced by ligation of the inferior mesenteric vein may lead to dysfunction of the intestinal mucosa. This phenomenon is similar to that of DSS-induced colitis. One study has shown that chronic inflammation in patients with UC leads to a reduction in mucosal vasodilation due to strong oxidative stress (Hatoum et al., 2003). Angiographic studies have suggested that colon blood vessels are twisted, dilated and distributed irregularly in the early stage of UC, and there is a decrease in the diameter of the injured vessels, blood vessel density and blood flow with further development of UC (Chidlow et al., 2007). Furthermore, confocal endoscopy has also confirmed that VP increases in colon mucosa of patients with UC (Tarnawski et al., 2009). Disturbance of microcirculation in colon tissue may result in slowed blood flow and increased VP, which leads to local tissue hypoxia because blood oxygen cannot be transported to the mucosal epithelial cells. So, increased VP may be an important factor in the pathogenesis of UC.

VEGF is one of the most potent angiogenic factors, which has specificity for vascular endothelial cells. VEGF is directly associated with angiogenesis and inflammation in human and experimental UC (Scaldaferri et al., 2009). It has been confirmed that VEGF induces an increase in VP in UC, especially in the early stage, and VEGF inhibition has been shown to decrease VP and prevent further development of UC (Tolstanova et al., 2009). It has been shown that lymphangiogenesis, a process also enhanced in IBD and driven by VEGF-C, plays a protective role in animal models of UC (D'Alessio et al., 2014). Another study (Koutroubakis et al., 2004) has shown that IBD patients with elevated levels of VEGF expression in blood, which also indicates that VEGF is involved in the angiogenesis and VP change in IBD. In the early stage of UC, the increased expression of VEGF in colonic tissue may be due to vascular destruction and increased HIF-1 expression (Trojanowska, 2010). These results support the idea that increased VP by VEGF precedes mucosal disorder in the early stage of UC.

HIF-1α is an intranuclear protein that is produced by a decreased level of intracellular oxygen. HIF-1α has been shown to be a major inducer of hypoxia-driven VEGF. Increased levels of HIF-1α in tissue specimens and high levels of VEGF in serum samples of UC patients have been reported (Tajdini et al., 2013). Likewise, expression of HIF-1α increased in colonic submucosal vascular endothelial cells, vascular smooth muscle cells and myenteric plexus neurons in the DSS-induced UC model. As one of the target genes regulated by HIF-1α, it has been shown that the expression of iNOS gene could be induced by HIF-1(Surh et al., 2001). Besides, many iNOS-positive cells also appear in the lamina propria of the colonic tissue of DSS induced UC (Saijo et al., 2015). The occurrence of iNOS-positive cells is associated with IBD activity (Beck et al., 2004). With the aggravation of inflammation, the expression of iNOS increases rapidly, which activates production of large amounts of NO and oxyradicals, causing tissue and cell damage. Beyond that, iNOS is the primary cause of increased synthesis of prostaglandins (PGs; mostly PGE2) when inflammation occurs. Excessive PGE2 and NO result in increased vasodilation and VP, thus leading to mucosal congestion and edema (Blouin et al., 2004), which contributes to the initiation and development of inflammation.

Most studies have shown that intestinal epithelial oxygen supply decreases in patients with IBD (Hatoum et al., 2005; Taylor and Colgan, 2007). Mice with mild colitis (early stage) are complicated with hypoxia and decreased hematocrit and vascular density. On the contrary, mice with severe colitis (late stage) have reduced hypoxia and increased hematocrit and vascular density (Harris et al., 2011). Hypoxia not only maintains or aggravates inflammation via stabilization of HIF-1α (Palazon et al., 2014), but also influences the local mucosal tissue pH (Mogi et al., 2009). Subsequently, an acidic environment is not only the result of inflammation, but also affects the degree and outcome of inflammation. In this study, severe hypoxia was observed in the early stage of DSS-induced colitis, which even extended to the lamina propria and submucosa. This is consistent with previous studies (Saijo et al., 2015). The pathological and microstructural observation of colon tissues revealed that submucosal vascular congestion, endothelial damaged, and platelet aggregation occurred before colonic epithelial destruction in the early stage of UC, which further demonstrated that colon tissue microcirculation disturbance co-existed with hypoxia and also occurred before damage to intestinal epithelial barrier function.

Expression of TNF-α and IL-6 in colonic tissue was significantly increased. This indicated the presence of hypoxia and cytokine imbalance in the early stage of UC, which can be explained as follows. (1) Vascular and lymphocyte hyperplasia and cell metabolic activities increase in the early stage of IBD (Neurath et al., 2001). (2) During the occurrence and development of IBD, many platelets are activated (Suzuki et al., 2001), and the types, number and bioactivities of glycoproteins on the platelet membranes change, which causes platelet adhesion and aggregation, thus increasing blood viscosity, vasoconstriction, and formation of micro-thrombi that may adversely influence intestinal mucosal microcirculation and aggravate intestinal mucosal hypoxia damage. (3) Obvious anoxia is observed in colonic mucosal tissues, which, with increased expression of HIF-1α and oxidative stress, could increase HIF-1α expression directly or indirectly by regulating matrix connective tissue cells releasing cytokines (Giatromanolaki et al., 2003). (4) Inflammatory factors like TNF-α and IL-6 increase significantly with enhanced activity in the early stage of UC (Fiocchi, 2004), which induces activation of HIF-1α (Albina et al., 2001). Tissue hypoxia induced by microcirculatory disturbance affects the occurrence, development or even prognosis of UC.

According to the results of LC-ESI-MS/MS, QCS contained multiple bioactive compounds, in which indirubin has significant anti-inflammatory activity (Gao et al., 2016). Notoginsenoside R1 and ginsenosides Rb1 and Rg1 are the primary active ingredients of Radix notoginseng. Notoginsenoside R1 down-regulates the increased expression of VEGF and matrix metalloproteinase-2, promotes regeneration of endothelial cells, and reduces the thickened extracellular matrix (Chen et al., 2004). Ginsenosides Rb1 and Rg1 activate and produce NO by regulating the PI3K/Akt/eNOS signal pathways and arginine transformation on endothelial cells, so as to increase the endothelium-dependent hemangiectasis in rats (Pan et al., 2012). Our recent study found that Panax notoginseng could promote repair of injuries of colonic mucosa and microvessels via downregulating VEGFA isoforms and inhibiting Rap1GAP/TSP1 signaling pathway (Wang et al., 2018). Gallic acid (Chang et al., 2015), a primary active ingredient of Galla chinensis, has multiple bioactivities like anti-inflammation, anti-mutation, anti-oxidation, and anti-free radicals. Portulaca oleracea (Zhou et al., 2015) possesses a wide spectrum of pharmacological properties such as neuroprotective, antimicrobial, antidiabetic, antioxidant, anti-inflammatory, antiulcerogenic, and anticancer activities. These may be why QCS has multi-pathway and multi-target therapeutic efficacy in the treatment of DSS-induced colitis.

Although this study proved that colonic VP increased earlier than EP in the early stage of colitis induced by DSS, damage of the vascular endothelial barrier occurred earlier than that of intestinal mucosa. The mechanisms of VP increase leading to EP increase in the early stage of colitis are still unclear and need to be investigated further. Meanwhile, a reasonable orthogonal design study on QCS ingredients is required to verify whether the clinical efficacy can be maximized by optimizing compatibility of prescription and regulating the dosage of a single drug.

In summary, QCS treatment could alleviate colonic tissue inflammatory, microvascular structural damage and local tissue hypoxia in DSS-induced colitis. All of these may be attributed to the improving effect of QCS on vascular endothelial barrier function by regulating the VEGF/HIF-1α signaling pathway, and therefore QCS could serve as alternative medicine for patients suffering from colitis.

## Data availability

All data generated or analyzed during this study are included in this published article.

## Author contributions

BYS and JY contributed equally to this work. HH designed the research and wrote the paper; BYS and JY performed the rat experiments; SW, WZ, RW, and BS performed biochemical analysis; JL and JS analyzed the data.

### Conflict of interest statement

The authors declare that the research was conducted in the absence of any commercial or financial relationships that could be construed as a potential conflict of interest.

## References

[B1] Albina J. E. Mastrofrancesco B. Vessella J. A. Louis C. A. Henry W. L. Reichner J. S. (2001). HIF-1 expression in healing wounds: HIF-1α induction in primary inflammatory cells by TNF-α. Am. J. Physiol. Cell Physiol. 281:C1971. 10.1152/ajpcell.2001.281.6.C1971 11698256

[B2] Alsadi R. Boivin M. Ma T. (2009). Mechanism of cytokine modulation of epithelial tight junction barrier. Front. Biosci. 14, 2765–2778. 10.2741/3413 PMC3724223 19273235

[B3] Beck P. L. Xavier R. Wong J. Ezedi I. Mashimo H. Mizoguchi A.. (2004). Paradoxical roles of different nitric oxide synthase isoforms in colonic injury. Am. J. Physiol. Gastrointest. Liver Physiol. 286, 137–147. 10.1152/ajpgi.00309.2003 14665440

[B4] Blouin C. C. Soucy G. M. Richard D. E. (2004). Hypoxic gene activation by lipopolysaccharide in macrophages: implication of hypoxia-inducible factor 1α. Blood 103:1124–1187. 10.1182/blood-2003-07-2427 14525767

[B5] Chang Y. J. Hsu S. L. Liu Y. T. Lin Y. H. Lin M. H. Huang S. J.. (2015). Gallic acid induces necroptosis via TNF-α signaling pathway in activated hepatic stellate cells. PLoS ONE 10:e0120713. 10.1371/journal.pone.0120713 25816210 PMC4376672

[B6] Chen S. W. Li X. H. Ye K. H. Jiang Z. F. Ren X. D. (2004). Total saponins of Panax notoginseng protected rabbit iliac artery against balloon endothelial denudation injury. Acta Pharmacol. Sin. 25, 1151–1156. 15339390

[B7] Chidlow J. H. Shukla D. Grisham M. B. Kevil C. G. (2007). Pathogenic angiogenesis in IBD and experimental colitis: new ideas and therapeutic avenues. Am. J. Physiol. Gastrointest. Liver Physiol. 293:G5. 10.1152/ajpgi.00107.2007 17463183

[B8] Dai Y. C. Tang Z. P. Ma G. T. Gong Y. P. Liu W. Zhang Y. L. (2010). A review of Qingchang Shuan for treatment of ulcerative colitis. J. Tradition. Chin. Med. 30, 237–240. 10.1016/S0254-6272(10)60049-0 21053635

[B9] D'Alessio S. Correale C. Tacconi C. Gandelli A. Pietrogrande G. Vetrano S.. (2014). VEGF-C–dependent stimulation of lymphatic function ameliorates experimental inflammatory bowel disease. J. Clin. Invest. 124, 3863–3878. 10.1172/JCI72189 25105363 PMC4151217

[B10] Fiocchi C. (2004). Inflammatory bowel disease: autoimmune or immune-mediated pathogenesis? Clin. Dev. Immunol. 11:195 10.1080/17402520400004201 15559364 PMC2486322

[B11] Floch M. H. (2011). Inflammatory bowel disease. J. Clin. Gastroenterol. 45, 141–170. 10.1097/MCG.0b013e31822be119

[B12] Gao W. Guo Y. Wang C. Lin Y. Li Y. Sheng T.. (2016). Indirubin ameliorates dextran sulfate sodium-induced ulcerative colitis in mice through the inhibition of inflammation and the induction of Foxp3-expressing regulatory T cells. Acta Histochem. 118, 606–614. 10.1016/j.acthis.2016.06.004 27396532

[B13] Giatromanolaki A. Sivridis E. Maltezos E. Papazoglou D. Simopoulos C. Gatter K. C.. (2003). Hypoxia inducible factor 1α and 2α overexpression in inflammatory bowel disease. J. Clin. Pathol. 56:209. 10.1136/jcp.56.3.209 12610101 PMC1769899

[B14] Hao Y. Nagase K. Hori K. Wang S. Kogure Y. Fukunaga K.. (2014). Xilei san ameliorates experimental colitis in rats by selectively degrading proinflammatory mediators and promoting mucosal repair. Evid. Based Compl. Alternat. Med. 2014:10. 10.1155/2014/569587 25120575 PMC4120479

[B15] Harris N. R. Carter P. R. Yadav A. S. Watts M. N. Zhang S. Kosloskidavidson M.. (2011). Relationship between inflammation and tissue hypoxia in a mouse model of chronic colitis. Inflamm. Bowel Dis. 17:742. 10.1002/ibd.21423 20878754 PMC3013240

[B16] Hatoum O. A. Binion D. G. Gutterman D. D. (2005). Paradox of simultaneous intestinal ischaemia and hyperaemia in inflammatory bowel disease. Eur. J. Clin. Invest. 35, 599–609. 10.1111/j.1365-2362.2005.01567.x 16178878

[B17] Hatoum O. A. Miura H. Binion D. G. (2003). The vascular contribution in the pathogenesis of inflammatory bowel disease. Am. J. Physiol. Heart Circ. Physiol. 285:H1791. 10.1152/ajpheart.00552.2003 14561675

[B18] Irkorucu O. Tascilar O. Karakaya K. Emre A. Ucan B. Bahadir B.. (2008). The effect of sildenafil on an animal model for ischemic colitis. Dig. Dis. Sci. 53, 1618–1623. 10.1007/s10620-007-0033-9 17932755

[B19] Johansson M. E. Gustafsson J. K. Sjöberg K. E. Petersson J. Holm L. Sjövall H.. (2010). Bacteria penetrate the inner mucus layer before inflammation in the dextran sulfate colitis model. PLoS ONE 5:e12238. 10.1371/journal.pone.0012238 20805871 PMC2923597

[B20] Jump R. L. Levine A. D. (2010). Mechanisms of natural tolerance in the intestine: implications for inflammatory bowel disease. Inflamm. Bowel Dis. 10, 462–478. 10.1097/00054725-200407000-00023 15475760

[B21] Koutroubakis I. E. Xidakis C. Karmiris K. Sfiridaki A. Kandidaki E. Kouroumalis E. A. (2004). Serum angiogenin in inflammatory bowel disease. Dig. Dis. Sci. 49, 1758–1762. 10.1007/s10620-004-9565-4 15628698

[B22] Kurashima Y. Amiya T. Nochi T. Fujisawa K. Haraguchi T. Iba H.. (2012). Extracellular ATP mediates mast cell-dependent intestinal inflammation through P2X7 purinoceptors. Nat. Commun. 3:1034. 10.1038/ncomms,2023 22948816 PMC3658010

[B23] Mankertz J. Schulzke J. (2007). Altered permeability in inflammatory bowel disease: pathophysiology and clinical implications. Curr. Opin. Gastroenterol. 23:379. 10.1097/MOG.0b013e32816aa392 17545772

[B24] Masakazu Y. (2014). Ulcerative colitis-associated colorectal cancer. World J. Gastroenterol. 20:16389 10.3748/wjg.v20.i44.16389 25469007 PMC4248182

[B25] Mogi C. Tobo M. Tomura H. Murata N. He X. D. Sato K.. (2009). Involvement of proton-sensing TDAG8 in extracellular acidification-induced inhibition of proinflammatory cytokine production in peritoneal macrophages. J. Immunol. 182:3243. 10.4049/jimmunol.0803466 19234222

[B26] Murthy S. N. Cooper H. S. Shim H. Shah R. S. Ibrahim S. A. Sedergran D. J. (1993). Treatment of dextran sulfate sodium-induced murine colitis by intracolonic cyclosporin. Dig. Dis. Sci. 38, 1722–1734. 10.1007/BF01303184 8359087

[B27] Neurath M. F. Finotto S. Fuss I. Boirivant M. Galle P. R. Strober W. (2001). Regulation of T-cell apoptosis in inflammatory bowel disease: to die or not to die, that is the mucosal question. Trends Immunol. 22:21. 10.1016/S1471-4906(00)01798-1 11286687

[B28] Palazon A. Goldrath A. Nizet V. Johnson R. S.. (2014). HIF transcription factors, inflammation, and immunity. Immunity 41, 518–528. 10.1016/j.immuni.2014.09.008 25367569 PMC4346319

[B29] Pan C. Huo Y. An X. Singh G. Chen M. Yang Z.. (2012). Panax notoginseng and its components decreased hypertension via stimulation of endothelial-dependent vessel dilatation. Vascul. Pharmacol. 56:150. 10.1016/j.vph.2011.12.006 22239978

[B30] Rezaie A. Parker R. D. Abdollahi M. (2007). Oxidative stress and pathogenesis of inflammatory bowel disease: an epiphenomenon or the cause? Dig. Dis. Sci. 52, 2015–2021. 10.1007/s10620-006-9622-2 17404859

[B31] Saijo H. Tatsumi N. Arihiro S. Kato T. Okabe M. Tajiri H.. (2015). Microangiopathy triggers, and inducible nitric oxide synthase exacerbates dextran sulfate sodium-induced colitis. Lab. Invest. 95, 728–748. 10.1038/labinvest.2015.60 25938626

[B32] Scaldaferri F. Vetrano S. Sans M. Arena V. Straface G. Stigliano E.. (2009). VEGF-A links angiogenesis and inflammation in inflammatory bowel disease pathogenesis. Gastroenterology 136, 585–595. 10.1053/j.gastro.2008.09.064 19013462

[B33] Surh Y. J. Chun K. S. Cha H. H. Han S. S. Keum S. Y. Park K. K. (2001). Molecular mechanisms underlying chemopreventive activities of anti-inflammatory phytochemicals: down-regulation of COX-2 and iNOS through suppression of NF-κB activation. Mut Res. 480–481, 243–268. 10.1016/S0027-5107(01)00183-X 11506818

[B34] Suzuki K. Sugimura K. Hasegawa K. Yoshida K. Suzuki A. Ishizuka K.. (2001). Activated platelets in ulcerative colitis enhance the production of reactive oxygen species by polymorphonuclear leukocytes. Scand. J. Gastroenterol. 36, 1301–1306. 10.1080/003655201317097164 11761021

[B35] Tajdini M. Mirbagheri S. A. Nikooie R. Ostovaneh M. R. Ghoreyshi Hefzabad S. M. Garg S. K. (2013). Tissue hypoxia in pathogenesis of ulcerative colitis: should we change all our beliefs? Gastroenterology 144:1487 10.3109/00365521.2013.845798 24134784

[B36] Tarnawski A. S. Coron E. Mosnier J. F. Ahluwalia A. Rhun M. L. Galmiche J. P. (2009). *In vivo* detection by confocal endomicroscopy of two distinct structural abnormalities in angioarchitecture and increased vascular permeability in colonic mucosa of patients with IBD in remission: mechanistic implications. Gastroenterology 136, A-112 10.1016/S0016-5085(09)60503-5

[B37] Taylor C. T. Colgan S. P. (2007). Hypoxia and gastrointestinal disease. J. Mol. Med. 85, 1295–1300. 10.1007/s00109-007-0277-z 18026919

[B38] Thornton M. Solomon M. J. (2002). Crohn's disease: in defense of a microvascular aetiology. Int. J. Colorectal Dis. 17, 287–297. 10.1007/s00384-002-0408-5 12172921

[B39] Tolstanova G. Deng X. French S. W. Lungo W. Paunovic B. Khomenko T.. (2012). Early endothelial damage and increased colonic vascular permeability in the development of experimental ulcerative colitis in rats and mice. Lab. Invest. 92:9. 10.1038/labinvest.2011.122 21894149

[B40] Tolstanova G. Khomenko T. Deng X. Chen L. Tarnawski A. Ahluwalia A.. (2009). Neutralizing anti-vascular endothelial growth factor (VEGF) antibody reduces severity of experimental ulcerative colitis in rats: direct evidence for the pathogenic role of VEGF. J. Pharmacol. Exp. Ther. 328:749. 10.1124/jpet.108.145128 19060224

[B41] Trojanowska M. (2010). Cellular and molecular aspects of vascular dysfunction in systemic sclerosis. Nat. Rev. Rheumatol. 6:453. 10.1038/nrrheum.2010.102 20585340 PMC3824624

[B42] Wang S. Tao P. Zhao L. Zhang W. Hu H. Lin J. (2018). Panax notoginseng promotes repair of colonic microvascular injury in sprague-dawley rats with experimental colitis. Evid. Based Compl. Alternat. Med. 2018, 1–8. 10.1155/2018/4386571 29785192 PMC5896412

[B43] Xiao H. T. Lin C. Y. Ho D. H. Peng J. Chen Y. Tsang S. W.. (2013). Inhibitory effect of the gallotannin corilagin on dextran sulfate sodium-induced murine ulcerative colitis. J. Nat. Prod. 76, 2120–2125. 10.1021/np4006772 24200352

[B44] Xin H. L. Yan-Feng X. U. Yue X. Q. Hou Y. H. Min L. I. Ling C. Q. (2008). [Analysis of chemical constituents in extract from Portulaca oleracea L.with GC-MS method]. Pharm. J. Chin. Peoples Liber. Army. 24, 133–136. (Chinese)

[B45] Xu W. Qiu X. Zhang J. Zhu D. Yang Y. Lu C. (2012). [Analysis of saponins in Panax notoginseng by UPLC-LTQ-Orbitrap MS/MS]. Yao Xue Xue Bao. 47, 773–778. (Chinese) 22919726

[B46] Zhou Y. X. Xin H. L. Rahman K. Wang S. J. Peng C. Zhang H. (2015). *Portulaca oleracea* L.: a review of phytochemistry and pharmacological effects. Biomed Res. Int. 2015:925631. 10.1155/2015/925631 25692148 PMC4321094

