# Supplementary material for: Qingchang Suppository Ameliorates Colonic Vascular Permeability in Dextran-Sulfate-Sodium-Induced Colitis
Source: Front Pharmacol. 2018 Oct 31;9:1235. doi: 10.3389/fphar.2018.01235 (PMC6220057; doi:10.3389/fphar.2018.01235)
Supplement: Supplementary file 1 [file Table_1.DOCX]

Supplementary Material

Qingchang Suppository ameliorates colonic vascular permeability in dextran-sulfate-sodium‑induced colitis

First Author*, Boyun Sun, Jianye Yuan

*** Correspondence:** Dr. Hongyi Hu, hongyihu2003@shutcm.edu.cn

# Supplementary Table

**Tab.1** Characterization and quantification of the major biochemical components from Qingchang Suppository by LC-ESI-MS/MS

| **Peak no.** | ***tR* (min)** | **Compound** | **[M-H]-**  *m/z* | **[M+HCOO] –**  *m/z* | **MS/MS date**  **(measured from [M-H]-)** | **Content**  **(mg/g)** *b* |
| --- | --- | --- | --- | --- | --- | --- |
| 1 | 1.12 | Ethyl gallate | 197.6500 |  | 163.10 |  |
| 2 | 1.37 | Gallic acid isomer | 169.0000 |  | 125.2 |  |
| 3 | 1.67 | Gallic acid *a* | 169.0000 |  | 125.2 | 11.358 |
| 4 | 3.35 | Methyl gallate *a* | 183.1500 |  | 124.3 | 0.610 |
| 5 | 5.57 | Notoginsenoside R1*a* |  | 977.4500 | 769.6, 619.6 | 6.482 |
| 6 | 5.77 | Ginsenoside Re *a* |  | 991.5500 | 799.6, 619.6 | 0.548 |
| 7 | 5.87 | Ginsenoside Rg1 *a* | 845.6000 |  | 799.5, 627.6 | 8.940 |
| 8 | 6.60 | Quercetin *a* | 301.1500 |  |  | 0.002 |
| 9 | 6.64 | Luteolin *a* | 285.2000 |  |  | 0.001 |
| 10 | 7.31 | Ginsenoside Rb1 *a* | 1108.55 |  | 459.4, 203.0 | 3.560 |
| 11 | 7.62 | Ginsenoside F3 | 769.6000 | 815.4500 | 637.7 |  |
| 12 | 8.31 | Ginsenoside F2 | 783.6000 |  |  |  |
| 13 | 10.00 | Kaempferide *a* | 299.2000 |  |  | 0.001 |
| 14 | 10.57 | Unknown | 362.0000 |  | 193.3 |  |
| 15 | 10.69 | Indirubin *a* | 261.0000 |  |  | 0.972 |
| 16 | 11.03 | Unknown | 265.4000 |  | 117.1 |  |
| 17 | 11.98 | Unknown | 313.4000 |  | 167.6 |  |
| 18 | 12.06 | α-Linolenic acid *a* | 277.3500 |  |  | 0.685 |
| 19 | 13.02 | Unknown | 418.1500 |  | 299.5, 257.3 |  |
| 20 | 14.22 | Unknown | 653.3500 |  | 285.2, 203.4 |  |
| 21 | 14.54 | Ethyl stearate | 311.3000 |  | 149.2 |  |
| *a* identified by reference standards;  *b* The content were quantified by using external standard and area normalization method. | | | | | | |
